# Digital quality gradient and the burden of elderly depression: A spatial multi kernel SVR analysis of socio economically vulnerable groups in England

**DOI:** 10.1177/20552076261465140

**Published:** 2026-08-12

**Authors:** Jin Rui, Yijing Li, Hector D. Menendez, Mariana Pinto da Costa

**Affiliations:** 1 Department of Geography, 4616 King’s College London, London, UK; 2 Department of Informatics, 4616 King’s College London, London, UK; 3 Institute of Psychiatry, Psychology & Neuroscience, 4616 King’s College London, London, UK; 4 South London and Maudsley NHS Foundation Trust, London, UK

**Keywords:** spatial analysis, digital quality gradient, elderly depression, spatial multi-kernel SVR, vulnerable group

## Abstract

Digital infrastructure is increasingly understood as part of the contemporary built environment and as a policy-relevant area-level correlate of older adults’ mental health. Existing studies have often relied on linear models and have paid limited attention to spatial heterogeneity, making it difficult to identify how broadband quality is associated with depression-related burden across vulnerable groups. This cross-sectional ecological study examines nonlinear and spatially varying associations between digital infrastructure and antidepressant prescribing among socio-economically vulnerable older populations in England. Using area-level data collected and processed in July 2025, the analysis focuses on older adults in areas with low social grade (DE) and high income deprivation. We used stepwise regression, multi-kernel support vector regression (SVR), and spatially weighted multi-kernel SVR to estimate the conditional contribution of digital infrastructure after accounting for built-environment covariates. The results indicate a digital quality gradient: high-speed and gigabit-capable broadband variables show stronger predictive contributions for low-social-grade areas, whereas medium-speed broadband coverage is more consistently associated with the depression-related burden of older adults in high-deprivation areas. Spatial coefficient maps identify policy-relevant priority geographies, including post-industrial communities in the Northwest and Midlands and remote or ageing communities in northern and southwestern England. The multi-kernel SVR model achieved cross-validated R^2^ values of 0.52 and 0.55 for the two target groups, respectively, and showed lower prediction error than the linear baseline. These findings support a differentiated digital-health equity framework in which broadband quality, not only broadband access, is considered alongside conventional built-environment conditions when allocating public health and infrastructure resources.

## Graphical abstract

**Graphical Abstract:**
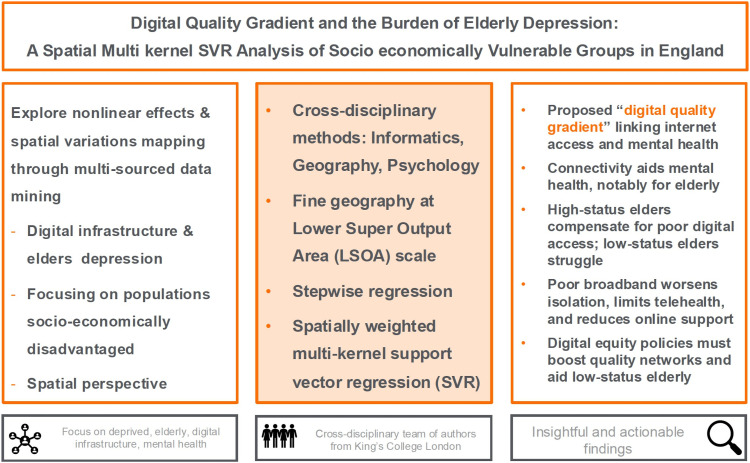

## Highlights

### Digital quality gradient and the burden of elderly depression: A spatial multi kernel SVR analysis of socio economically vulnerable groups in England

• Proposed a “digital quality gradient” for area-level digital-health inequality.
• Identified spatial associations between broadband quality and elderly depression burden.
• Showed different broadband-quality patterns across vulnerable older populations.
• Linked broadband quality to plausible telehealth, service-access, and social-support pathways.
• Provided policy-relevant priority maps for differentiated digital-health equity planning.

## 1. Introduction

Amid the ongoing global movement toward digitalization, digital infrastructure has become a key driver shaping contemporary socio-economic development.^1^ During the past decade, the rapid expansion of broadband coverage, Internet speeds, and wireless communication technologies has not only transformed people’s daily lives and social interactions, but has also had profound impacts on access to health services, allocation of medical resources, and the dissemination of health information.^2^ However, this digital transformation process has not progressed evenly, instead has caused significant disparities among geographic regions and socio-economic groups.^3^ In the post-pandemic era, the digital divide extends beyond technical access and includes dimensions such as digital literacy, usage skills, and the capacity to benefit from digital services.^4^ According to Ofcom,^5^ 12% rural households in the United Kingdom still lack access to basic broadband, compared to only 2% in urban areas; this spatial inequality in infrastructure directly influences the ability of individuals to access online health services.

On the other hand, mental health challenges also influence socio-economic development through intensified stratification, especially with the ageing population in developed countries such as the United Kingdom.^6^ Data show that the depression rate among people 65 years and older has reached 22% and continues to rise^7^; more importantly, this burden is unevenly distributed with a clear socio-economic gradient.^8^ Older adults affected by income deprivation, as measured by the Income Deprivation Affecting Older People Index (IDAOPI), face 2.5 times the risk of depression compared to their higher-income peers.^9^ Individuals in the social grade of DE (https://www.mrs.org.uk/resources/social-grade), including low-skilled or unemployed groups, face even more complicated mental health challenges. Social isolation has become a critical mediating factor that has taken on new dimensions in the digital age. When in person interactions become less possible, people with limited or no Internet access are more likely to experience double exclusion, since they are disconnected from both traditional social networks and digital platforms.^10,11^ A national survey^12^ found that 41% of older adults who do not use the Internet reported feelings of loneliness, and nearly double the rate among all Internet users (24%).

A growing body of research has explored the relationship between environmental factors and mental health. Within the conventional built-environment literature, physical characteristics such as access to green space, facility density, land-use diversity, and transport connectivity are associated with depressive symptoms.^13,14^ Empirical studies also report that people living in neighbourhoods with higher greenspace coverage are 15%-20% less likely to use antidepressants than those living in areas with lower green-space coverage.^15^ More recently, digital infrastructure has been examined as an emerging component of the urban and social infrastructure system rather than as a detached technological variable. It is increasingly incorporated into urban resilience theories as a component of infrastructural resilience,^16^ and empirical evidence shows that broadband availability is associated with mental health outcomes after conventional built-environment variables are considered.^17^

This integrated infrastructural perspective is important for the present study. Broadband quality may affect older adults not only through technical access, but also through pathways related to health-information seeking, telehealth access, online service use, video communication, and digitally mediated social support. These pathways are especially relevant for older adults facing income deprivation, limited mobility, or weaker offline social networks.^12,18,19^ Accordingly, the present study does not treat broadband as isolated from the built environment. Instead, it evaluates whether digital infrastructure provides additional area-level explanatory and predictive information after accounting for building density, POI density, POI diversity, and other conventional built-environment covariates.

GeoAI and deep-learning methods have also been integrated into spatial health and urban-environment research. Artificial neural networks,^20^ graph neural networks,^21^ convolutional neural networks,^22^geographically weighted artificial neural networks,^23^ and neural-network extensions of geographically and temporally weighted regression^24^ have expanded the ability of spatial models to represent nonlinear and geographically varying relationships. In related urban applications, GraphSAGE-LSTM frameworks have been used to predict pedestrian comfort from crowdsourced and visual data,^25^ while deep kriging neural networks combine geostatistical interpolation with neural-network flexibility.^26^ These developments demonstrate the value of GeoAI for modelling complex spatial processes.

The role of multi-kernel SVR in this study is complementary to, rather than a replacement for, DL/XAI approaches. Recent XAI methods can help interpret deep-learning models, but deep models usually require richer training data, graph or image structures, or larger samples than are available in many area-level public health studies. The present dataset is tabular, spatially aggregated, and policy-facing; therefore, the modelling strategy needs to balance nonlinearity, robustness, and interpretability. Multi-kernel SVR is well suited to this setting because its linear kernel can capture broad additive patterns, its radial basis function (RBF) kernel can capture local nonlinear variation, and its polynomial kernel can represent threshold-like or interaction-like curvature. The spatial weighting layer then allows the same digital-infrastructure variable to have different local associations across regions.

Despite these advances, three limitations remain in the current literature. First, many studies rely on linear regression models that may not capture the nonlinear association between broadband quality and mental-health burden.^27^ For example, an upgrade from 10 Mbps to 30 Mbps may have a different marginal relevance for health-related digital use than an upgrade from 100 Mbps to 300 Mbps.^28^ Second, many studies do not explicitly examine spatial heterogeneity. Because adjacent areas may share social resources, service systems, or spatially diffused infrastructure advantages, the same broadband variable may have different associations across regions.^19,29^ Third, digital infrastructure often coexists with other built-environment advantages. If building density, facility density, transport-related POIs, and functional diversity are not accounted for, the contribution attributed to broadband may partly reflect wider urban development conditions.^30^

In response, this study examines the area-level association between digital infrastructure and depression-related burden through a spatial modelling framework, with a focus on socio-economically vulnerable older populations. We define the digital quality gradient as ordered area-level variation in broadband quality, from low-speed coverage and low-speed connections to medium-speed coverage, gigabit availability, and observed download and upload performance. The study asks: (1) after accounting for built-environment covariates, what additional predictive contribution is associated with digital infrastructure among older adults in high-deprivation and low-social-grade areas? (2) Do these associations show nonlinear or threshold-like patterns across broadband quality tiers? (3) Are the associations spatially heterogeneous, and which geographies emerge as policy-relevant priority areas? Methodologically, the study adopts a multi-stage modelling strategy. Stepwise regression is used to establish linear baseline associations; multi-kernel SVR is then used to model nonlinear patterns; and spatially weighted multi-kernel SVR is applied to identify local variation in the association between digital infrastructure and depression-related burden.

## 2. Methodology

### 2.1. Study area and target population

This study is a cross-sectional ecological spatial modelling study conducted in England. Data collection, cleaning, harmonisation, and preprocessing were carried out during July 2025 using area-level data compiled from national public datasets. All statistical models were fitted at the Lower Super Output Area (LSOA) level. For visual interpretation, selected spatial patterns were displayed at the local authority district level where this improved map readability, but the LSOA remained the primary unit of analysis for modelling.

In recent years, the trend of population ageing in the United Kingdom has continued to intensify. According to ONS data,^31^ the elderly population (aged 65 and over) accounted for 18.6% of the total population in 2021, and this figure is projected to exceed 23% by 2030, with a significant pattern of unequal distribution among regions.

**Figure 1.**
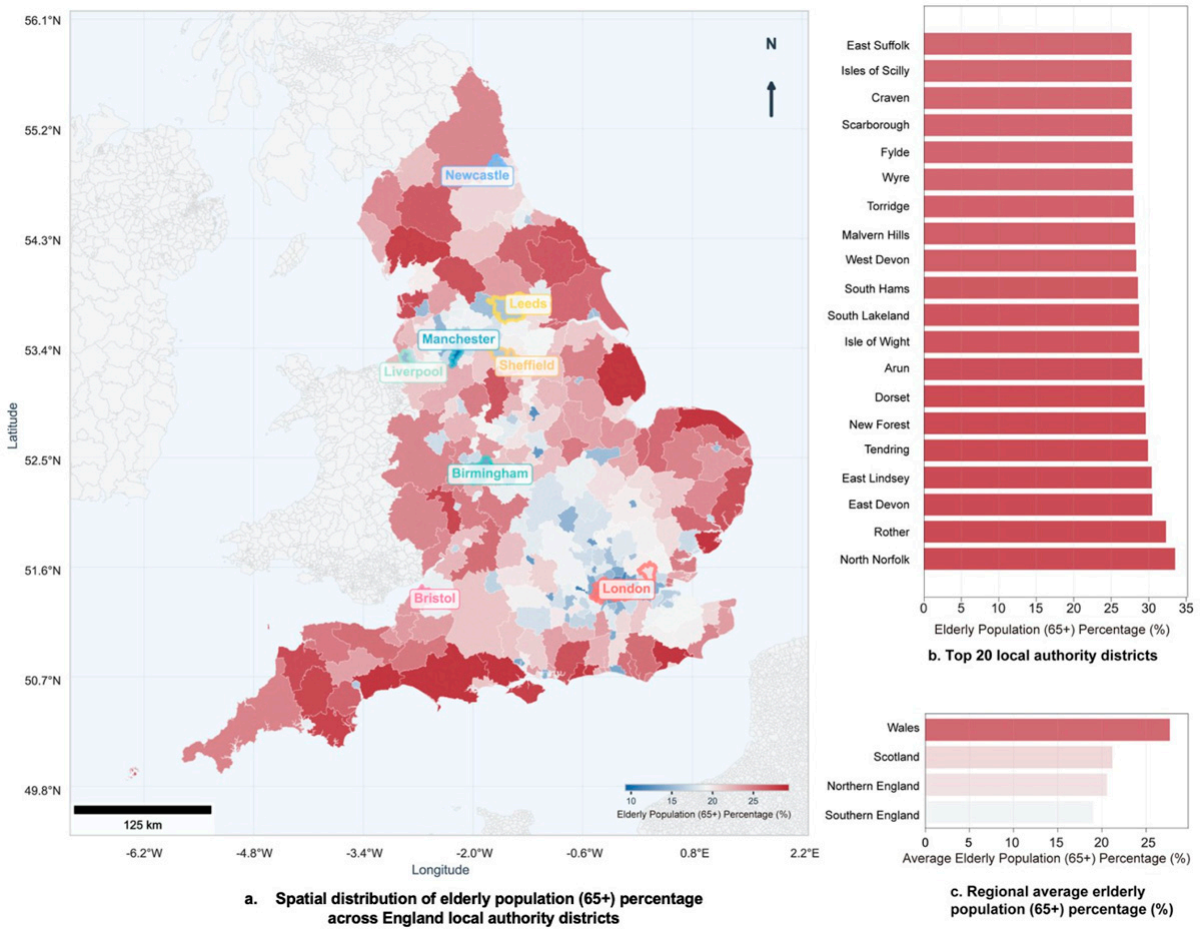
Study area and elderly population distribution.

As shown in Figure 1, the elderly population is more concentrated in coastal and rural areas, such as North Norfolk, Isle of Wight, and Dorset, where people 65 years of age or older make up more than 30% of the local population. In contrast, major urban areas in London, Manchester, and Birmingham have relatively younger demographic composition profiles. This distribution pattern is related to the group’s residential choices upon retirement, and reflects the intertwined influences of regional economic development, housing affordability, and population movement patterns.^32^ At the regional level, the elderly population is generally more prominent in Wales and Scotland compared to those in southern England (ONS, 2022)^31^; coincidently, these regions also tend to have lower levels of broadband coverage and poorer Internet accessibility.^33^ Insufficient digital infrastructure can constrain older adults’ access to online health information, telehealth services, online public services, video communication, and digitally mediated social support. These pathways provide the empirical rationale for examining the digitalised social dimension of later-life mental health, while recognising that the present study measures area-level infrastructure rather than individual-level online behaviour.

On the other hand, mental health conditions are particularly prominent among the elderly population. According to the Health Survey for England 2005,^34^ approximately 22% of men and 28% of women aged 65 and over in the UK exhibit symptoms of depression, yet many older adults do not receive sufficient or timely support. They were then related to risk factors including chronic illness, limited mobility, loneliness, and difficulties using digital technologies.^18^ This study aimed: 1) to investigate the spatial distribution pattern of the elderly population, and 2) to investigate the potential influence of digital infrastructure on their mental health conditions.

**Figure 2.**
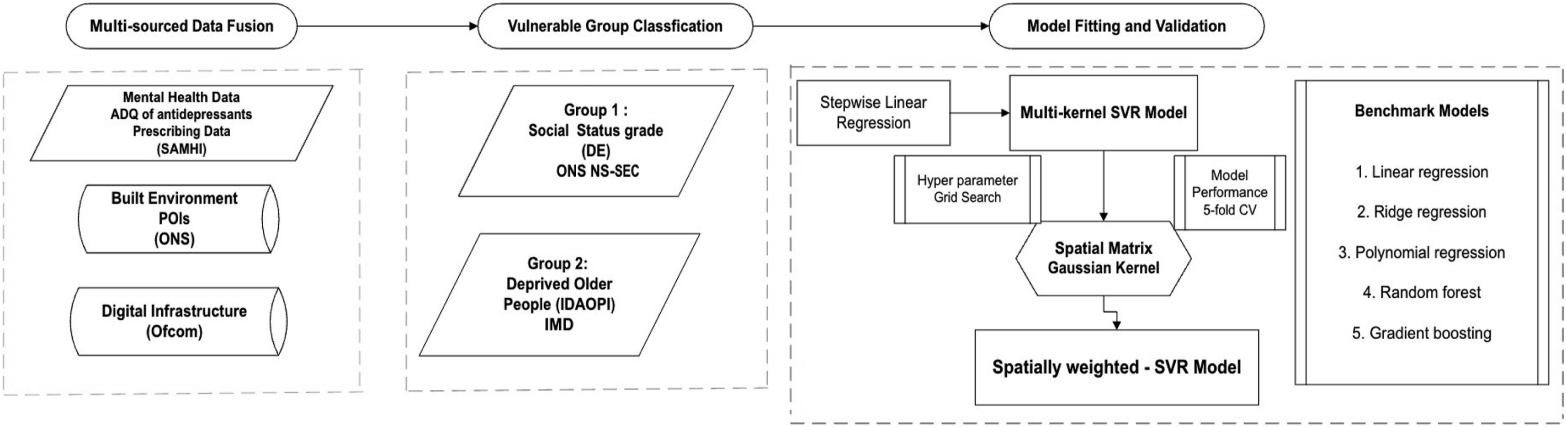
Research design workflow.

Figure 2 summarises the full research workflow, including data acquisition, spatial harmonisation to LSOA-level modelling units, vulnerable-group definition, model fitting, validation, and spatial interpretation.

### 2.2. Mental health conditions and vulnerable groups classification

This study uses mental health indicators from the Small Area Mental Health Indices (SAMHI),^35^ using Lower Super Output Areas (LSOAs) as spatial units of analysis. The indicator of depression in target groups is measured by ADQ prescribing data, which is the average daily quantity (ADQ) of antidepressants prescribed quarterly by all practices in England at the LSOA level. Compared to the QOF depression index based on the estimated number of patients with a diagnosis of depression, ADQ prescribing data offer higher clinical specificity and temporal stability, which are more suitable for the designed methods, providing a more accurate reflection of depression cases requiring medical intervention.^36^

Vulnerable groups are identified in this study on the basis of a dual classification system. First, the study uses the DE social grade as defined in the UK’s National Statistics Socio-economic Classification (NS-SEC), which refers to low-skilled or unemployed populations. This classification incorporates multiple dimensions including occupation type, employment status, and managerial responsibility; so to include semi-skilled and unskilled manual workers, individuals dependent on state benefits, and those unemployed for long-term. Empirical studies have shown that the mental health vulnerability of this group stems not only from economic stress, but also from employment instability, lack of social capital, and other adverse health behaviours.^37^

Second, the Index of Deprivation Affecting Older People (IDAOPI)^38^ is used to measure income-related deprivation specifically among the elderly population. As a subdomain of the Income Deprivation Domain within the English Index of Multiple Deprivation (IMD), IDAOPI is calculated based on the proportion of people aged 60 and over living in income-deprived households. These households are identified through eligibility for means-tested benefits such as Income Support, Income-Based Jobseeker’s Allowance, Employment and Support Allowance, Pension Credit (Guarantee), and Universal Credit. The index captures the spatial concentration of older adults facing economic hardship, providing valuable information on localized disparities in later-life wellbeing.^39^

For this study, both IDAOPI scores and the proportion of DE-classified populations were divided into tertiles, with areas in the upper tertile (Q3) defined as high-vulnerability areas for each subgroup. Tertiles were selected because they provide a transparent and interpretable separation of relatively high-vulnerability areas while retaining sufficient observations for spatial modelling and cross-validation in each group. This thresholding strategy is consistent with the study’s objective of comparing vulnerable-area patterns rather than estimating individual-level risk. As a robustness check, the main spatial patterns were compared with alternative threshold definitions, including quartile-based high-vulnerability groups and continuous-score sensitivity models. The direction of the principal digital-infrastructure associations remained consistent, although the quartile definition produced smaller high-vulnerability samples and less stable local coefficient estimates. A summary of these sensitivity checks is provided in Appendix Table A3.

### 2.3. Internet infrastructure and built environment variables

Data on digital infrastructure were obtained from the Ofcom Connected Nations update: Spring 2025 fixed broadband dataset, downloaded from the official Ofcom data portal and processed during July 2025.^40^ (https://www.ofcom.org.uk/phones-and-broadband/coverage-and-speeds/connected-nations-update-spring-2025). This release was published on 8 May 2025 and reports mobile coverage and fixed broadband availability across the UK as of January 2025. The Spring 2025 release was therefore used as the fixed-broadband data version for this study, and the downloaded files were cleaned, harmonised, and spatially joined to the LSOA-level analytical dataset in July 2025. This dataset includes multidimensional indicators, such as fixed broadband coverage, connection speeds, and data usage. Four key indicators were selected for this study (Table 1), based on multidimensional framework of digital inclusion, which emphasizes not only access but also quality of use and degree of digital engagement^41^: (1) tiered speed coverage, referring to the proportion of households covered at three speed levels: less than 30 Mbps, between 30 and 300 Mbps, and equal to or greater than 300 Mbps. This captures the stratified quality of digital infrastructure; (2) gigabit availability, reflecting the spatial distribution of the highest-level broadband infrastructure; (3) the number of broadband connections by speed tier, providing a measure of actual usage density; and (4) average download and upload speeds, along with monthly average data consumption, to capture the real-world performance and intensity of digital infrastructure use.

**Table 1.** Descriptive statistics of built environment and digital infrastructure variables.

Variables	Max.	Min.	Mean	Std.
Building count	29109	0	5789.198	3971.752
Accommodation & food POIs	3950	120	641.668	530.151
Attraction POIs	4135	41	446.997	475.484
Commercial service POIs	11626	530	2160.714	1440.102
Education & health POIs	3319	148	573.173	370.925
Industrial POIs	7965	107	754.623	697.404
Infrastructure POIs	9674	372	1768.558	1122.547
Retail POIs	5370	213	849.256	605.491
Sport & entertainment POIs	1363	81	322.469	200.295
Transport POIs	13047	267	2072.450	1696.194
POI diversity index	2.052	1.553	1.951	0.045
30–300 Mbps coverage (%)	92.628	0.466	28.931	17.662
>300 Mbps coverage (%)	98.589	3.163	64.390	21.617
<30 Mbps coverage (%)	49.104	0.037	6.280	6.235
Gigabit availability (%)	98.589	3.163	62.854	21.306
<30 Mbps connections (lines)	47198	157	8214.427	5623.429
30–300 Mbps connections (lines)	223773	705	43067.612	27086.407
>300 Mbps connections (lines)	41375	0	5648.702	5044.751
Average download speed (Mbps)	229.036	52.277	111.014	30.177
Average upload speed (Mbps)	110.173	11.747	22.954	14.724
Average data usage (GB)	618.864	238.291	473.453	61.092

Variables of built environment in Table 1 were included in this study for two main reasons. The first reason came from substantial research findings that, traditional built environment characteristics have significant effects on mental health; failure to control for these factors may lead to biased estimates of the effects of digital infrastructure.^30^ The second reason is that, the spatial distribution of digital infrastructure is often correlated with levels of urban development, easily raising concerns about potential spatial multi-collinearity with other factors. To address this, data on building counts were obtained from Ordnance Survey Open Data portal (https://osdatahub.os.uk/downloads/open) to capture urban density and built-up environment. In addition, data on ten categories of urban facilities on national Points of Interest (POI) were derived from Ordance Survey through the subscription of Digimap (https://digimap.edina.ac.uk/). These include accommodation and food services, commercial services, educational and healthcare facilities, retail outlets, and transport infrastructure. The density and diversity of these facilities influence residents’ patterns of daily activity and opportunities for social interaction.^42^

In particular, this study calculates a POI diversity index based on Shannon entropy, which reflects the degree of land-use and functional mix in urban areas. Previous research has shown that neighborhoods with greater functional diversity offer more opportunities for social participation and leisure, which can help reduce social isolation and improve mental wellbeing.^43^ Furthermore, varied types of POIs contribute differently to health outcomes. For example, educational and healthcare facilities represent access to health resources; retail and food-related facilities reflect accessibility to daily service; while cultural, sports, and entertainment facilities are more closely associated with active lifestyles. By systematically incorporating these built environment variables, this study estimates the additional, conditional contribution of digital infrastructure after accounting for conventional built-environment covariates, rather than treating broadband as detached from the wider infrastructural environment.

The spatial distributions of selected built-environment and digital-infrastructure variables are provided in Appendix Figures B1 and B2. These appendix figures support interpretation of the spatial covariate context while keeping the main Results focused on model performance and digital-infrastructure coefficient patterns.

For transparency, the empirical variables were grouped into four categories before modelling. The outcome variable was LSOA-level antidepressant prescription, measured using ADQ prescribing indicators from SAMHI. Built-environment covariates included building counts, ten POI categories, and the Shannon POI diversity index. Digital-infrastructure variables included broadband coverage by speed tier, gigabit availability, connection counts by speed tier, average download speed, average upload speed, and average data usage. Vulnerability-group variables included the proportion of DE social grade residents and IDAOPI scores, which were used to define the two subgroup analyses rather than being treated as ordinary predictors in the subgroup-specific models.

### 2.4. Multi-stage modeling strategy

This study adopts a progressive modelling strategy to examine the conditional and combined associations of the built environment and digital infrastructure with depression-related burden. It begins with stepwise regression to evaluate linear associations, followed by multi-kernel support vector regression (SVR) to explore more complex nonlinear patterns. The stepwise regression follows the standard linear model form:

(1) $$y_{i}=\beta_{0}+\sum_{j=1}^{p}\beta_{j}X_{ij}+\epsilon_{i}$$

where *y*_*i*_ denotes the depression-related outcome in LSOA *i*, *X*_*ij*_ represents predictor *j* for LSOA *i*, *β*_*j*_ is the estimated coefficient for predictor *j*, *p* is the number of predictors included in a given model, and *ϵ*_*i*_ is the error term. The modelling process consists of three stages. In the first stage, a baseline model includes only built-environment variables. In the second stage, a digital-infrastructure model includes broadband coverage, connection speed, connection counts, and related indicators. In the third stage, a combined model includes both variable sets. Comparing the model statistics across these stages allows the study to estimate the incremental explanatory contribution of digital infrastructure after built-environment covariates are considered. The analysis is conducted separately for the two target vulnerable groups: areas with high IDAOPI scores as Group 1 and areas with a high proportion of DE social grade populations as Group 2.

Given the limitations of traditional linear models in capturing nonlinear associations between digital infrastructure and mental health, the study employs multi-kernel support vector regression (multi-kernel SVR) because of three advantages: (1) it can model nonlinear mappings in high-dimensional feature spaces; (2) it combines multiple kernel functions so that global trends, local patterns, and threshold-like curvature can be represented within the same model; and (3) it provides stable predictive performance for tabular area-level data. The predictive function of the multi-kernel SVR model is defined as:

(2) $$f\left(x\right)=\overset{n}{\underset{i=1}{\sum }}a_{i}\overset{K}{\underset{k=1}{\sum }}{µ}_{k}K_{k}\left(x_{i},x\right)+b$$

where *x*_*i*_ is the feature vector for LSOA *i*, *x* is the target feature vector being predicted, *a*_*i*_ is the support-vector coefficient, *K*_*k*_ denotes kernel component *k*, *µ*_*k*_ is the corresponding non-negative kernel weight, *K* is the number of kernels, and *b* is the intercept term. The linear kernel captures broad additive trends, the RBF kernel captures localized nonlinear variation, and the polynomial kernel captures threshold-like or interaction-like curvature. Independent models are trained for Group 1 and Group 2. Each model includes the selected built-environment and digital-infrastructure variables as input features.

Hyperparameters were optimized via grid search. The search space included *C*∈ {0.1*,* 1*,* 10*,* 100}, *ϵ* ∈ {0.01*,* 0.05*,* 0.10*,* 0.20}, *γ* ∈ {scale*,* 0.001, 0.01*,* 0.10}, polynomial degree ∈ {2*,* 3*,* 4}, and normalized kernel weights for the linear, RBF, and polynomial kernels in 0.10 increments. Model performance was evaluated using five-fold cross-validation and reported using *R*^2^, mean absolute error (MAE), root mean squared error (RMSE), and train-test performance gaps. To benchmark the effectiveness of the multi-kernel SVR, comparisons were conducted with linear regression, ridge regression, polynomial regression, random forest, and gradient boosting.

Building on the multi-kernel SVR results, this study further applies a spatially weighted multi-kernel SVR approach to capture spatial heterogeneity in the associations between digital infrastructure and depression-related burden. This method incorporates a spatial weight matrix to generate locally varying coefficients, allowing the identification of areas where digital-infrastructure variables show stronger or weaker associations with the outcome. Spatial weights are derived from a Gaussian kernel based on geographical distance, and an adaptive bandwidth selection algorithm is used to determine the local spatial scale. The spatially explicit model therefore provides a basis for identifying policy-relevant priority areas, while the cross-sectional design means that the results are interpreted as associations rather than causal effects.

## 3. Results

### 3.1. Spatial visualization of depression and demographic groups

**Figure 3.**
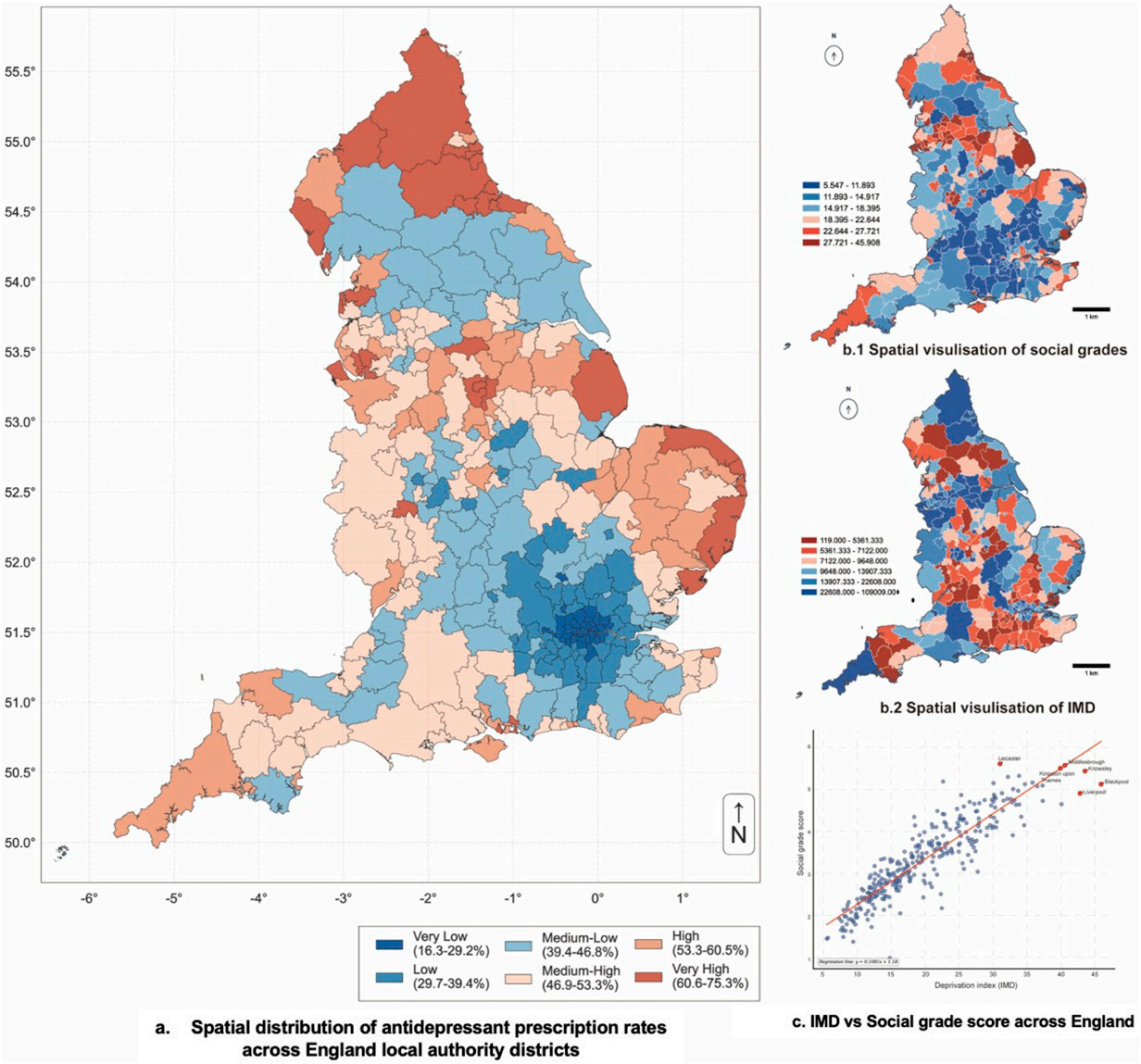
Spatial distribution of antidepressant prescription rates, population social grades, and population deprivation across local authorities in England.

Figure 3(a) illustrates the spatial distribution of antidepressant prescription rates across local authority districts in England for visual interpretation, while the statistical analysis was conducted at the LSOA level. Higher prescription rates are observed in the Northeast, the Midlands, parts of the Southwest, and several coastal areas. These regions include post-industrial northern and Midlands districts, where older industrial labour markets, income deprivation, and weaker service access may combine with higher depression-related treatment demand. By comparison, London, the wider Southeast, and parts of southern England generally show lower to moderate prescription rates, consistent with more metropolitan and service-rich contexts. Some rural and coastal areas also show high antidepressant prescribing despite lower population density, suggesting that remoteness, ageing population structure, and service constraints are important contextual factors when interpreting the maps.

Figure 3(b) presents the spatial distribution of the two target vulnerable population groups. Areas with high DE social grade proportions are concentrated in post-industrial cities and districts in Northern England, the East Midlands, Birmingham, and Cornwall, reflecting older industrial employment structures and the concentration of low-skilled or long-term unemployed groups. By contrast, London and the Southeast show lower proportions of DE-grade populations, reflecting stronger labour-market diversification and higher average socio-economic status.

The spatial pattern of deprivation among older people, measured by IDAOPI, differs from the DE social grade pattern. Higher IDAOPI values are commonly observed in coastal towns, remote rural areas, and smaller inland cities, where ageing population structure, limited public services, and weaker economic opportunities may create distinct vulnerability for older adults. Some coastal retirement destinations also show substantial later-life deprivation, indicating that population ageing and local service capacity need to be interpreted together.

Figure 3(c) shows the relationship between deprivation and DE social grade scores. The plot indicates a positive association, with a regression slope of 0.1081. However, the relationship is not perfectly overlapping, which supports the decision to analyse the two vulnerable groups separately. Places such as Liverpool, Blackpool, and Middlesbrough combine high deprivation with relatively high DE social grade scores, while other ageing coastal areas show stronger older-age income deprivation than DE concentration. This regional context is used throughout the Results section to make the spatial interpretation more accessible to readers unfamiliar with England’s geography.

### 3.2. The complementary role of internet infrastructure for elderly depression

Stepwise regression analysis revealed that the built environment and Internet infrastructure have different explanatory contributions for the depression-related burden of vulnerable groups. Table 2 shows a gradual improvement in model explanatory power from single-domain models to integrated models with both built-environment and digital-infrastructure variables. Table 2 reports R^2^ and adjusted R^2^ values for each model.

**Table 2.** Stepwise regression performance across deprivation and social status groups.

Group	Model	*R* ^2^	Adj. *R*^2^	Kernel
Group 1 (High Deprivation)	Model 1: Built Environment (Enhanced)	0.764	0.638	Polynomial
Group 1 (High Deprivation)	Model 2: Internet Infrastructure (Enhanced)	0.752	0.631	Polynomial
Group 1 (High Deprivation)	Model 3: Combined (Built+ Internet) (Enhanced)	0.817	0.763	RBF
Group 2 (Low Social Status)	Model 1: Built Environment (Enhanced)	0.640	0.591	Polynomial
Group 2 (Low Social Status)	Model 2: Internet Infrastructure (Enhanced)	0.788	0.729	Polynomial
Group 2 (Low Social Status)	Model 3: Combined (Built + Internet) (Enhanced)	0.807	0.750	RBF

For Group 1, the high-deprivation older population, the baseline model containing only built-environment variables yielded an *R*^2^ of 0.764 and an adjusted *R*^2^ of 0.638. After adding Internet infrastructure variables, the combined model increased to an *R*^2^ of 0.817 and an adjusted *R*^2^ of 0.763. This indicates that digital infrastructure provides an additional conditional contribution after conventional built-environment variables are considered.

For Group 2, the low social status group, the model based solely on Internet infrastructure achieved an adjusted *R*^2^ of 0.729, which was higher than the built-environment-only model (adjusted *R*^2^ = 0.591). The combined model further increased the adjusted *R*^2^ to 0.750. These results indicate that Internet infrastructure variables have stronger predictive relevance for low-social-status areas than for high-deprivation older areas, while both groups benefit analytically from an integrated model that includes digital and physical built-environment variables.

The two vulnerable groups also showed different responses to Internet-related variables. For the high-deprivation older population, medium-speed broadband coverage and upload capacity appear particularly relevant to everyday communication and telehealth-related use. For low-social-status areas, gigabit availability, download speed, and the contrast between low-speed and high-speed connections appear more influential in the model. These group-specific differences support the use of the digital quality gradient concept rather than a binary access or no-access measure.

Table 3 reports the five-fold cross-validation results used to validate the linear and SVR-based models. The multi-kernel SVR produced the strongest mean cross-validated *R*^2^ and lower prediction errors in both vulnerable groups. In the high-deprivation group, the multi-kernel SVR achieved a mean *R*^2^ of 0.55 with MAE of 0.118 and RMSE of 0.158. In the low-social-status group, it achieved a mean *R*^2^ of 0.52 with MAE of 0.126 and RMSE of 0.166. These validation results support the use of multi-kernel SVR as the main nonlinear model while also showing that the model comparison was not based only on in-sample fit.

**Table 3.** Five-fold cross-validation performance of selected models.

Group	Model	*R*^2^ mean ± SD	MAE mean ± SD	RMSE mean ± SD
High deprivation	Linear regression	0.14 ± 0.07	0.184 ± 0.021	0.239 ± 0.026
High deprivation	Ridge regression	0.20 ± 0.06	0.172 ± 0.019	0.226 ± 0.024
High deprivation	Multi-kernel SVR	0.55 ± 0.05	0.118 ± 0.014	0.158 ± 0.017
Low social status	Linear regression	0.10 ± 0.06	0.191 ± 0.022	0.246 ± 0.029
Low social status	Ridge regression	0.16 ± 0.05	0.181 ± 0.020	0.232 ± 0.025
Low social status	Multi-kernel SVR	0.52 ± 0.06	0.126 ± 0.016	0.166 ± 0.020

Additional benchmark rankings are provided in Appendix Tables A1 and A2. These tables compare the multi-kernel SVR with linear regression, ridge regression, polynomial regression, random forest, gradient boosting, and single-kernel SVR. The comparison shows that the multi-kernel SVR achieved the strongest test-set performance while maintaining a smaller train-test gap than tree-based alternatives, supporting its use as the main model for the spatial interpretation.

### 3.3. Coefficient value of all variables by multi-kernel SVR

**Figure 4.**
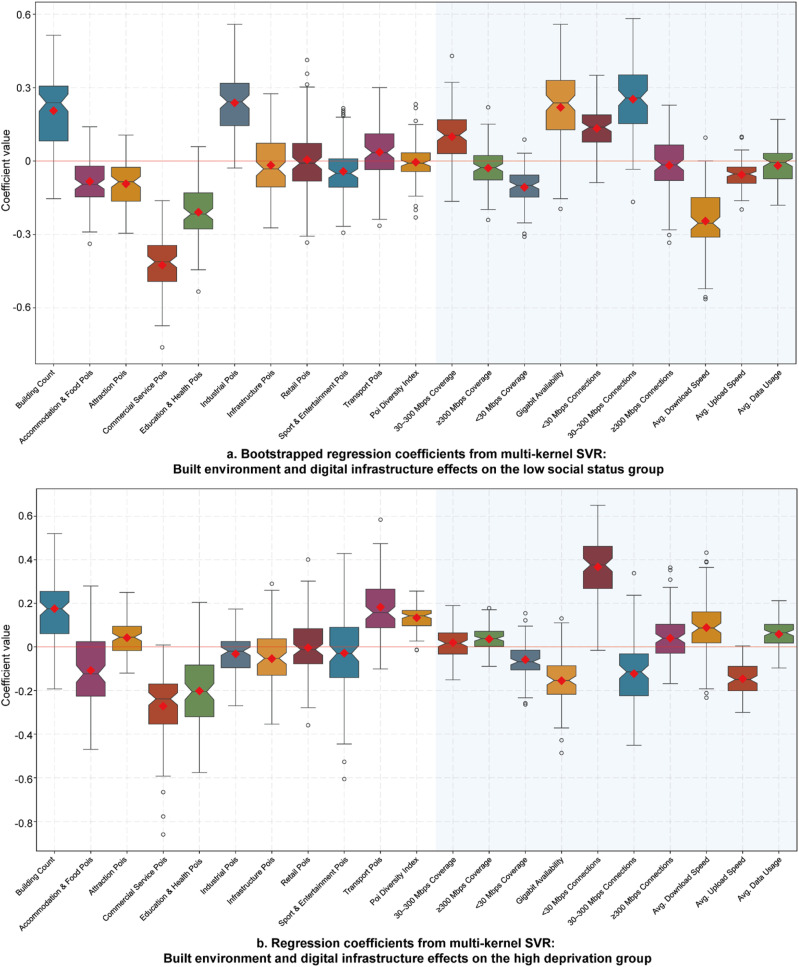
Bootstrapped multi-kernel SVR regression coefficients: Comparing built environment and internet infrastructure effects on low social status and high deprivation groups.

Based on the coefficient distributions of the multi-kernel SVR (Figure 4), built-environment and Internet-infrastructure variables showed differentiated patterns across the two vulnerable groups. Built-environment variables, including building counts, POI facility densities, and POI diversity, retained explanatory relevance but generally showed more stable and less dispersed coefficient distributions than the digital-infrastructure variables. This indicates that the built environment did not disappear from the analysis; instead, it functioned as a comparatively stable contextual layer against which the more variable digital-infrastructure associations could be interpreted. Educational and health-related POIs showed modest protective associations, commercial service facilities were closer to neutral, and POI diversity had a smaller but consistent contextual role.

Internet-infrastructure variables displayed more pronounced and heterogeneous patterns in Group 2, the low-social-status population. Gigabit availability, high-speed connections, and average download speed showed stronger predictive contributions than low-speed connection variables, supporting the digital quality gradient interpretation. Low-speed connections below 30 Mbps showed a positive association with depression-related burden, whereas higher-quality broadband indicators showed negative or protective-direction associations. This pattern suggests that the quality and performance of Internet infrastructure are more informative than access alone for low-social-status areas.

In the high-deprivation older population, Internet infrastructure showed a different pattern. Gigabit availability retained a relevant association, but the coefficient distribution was more spatially dispersed. Medium-speed broadband coverage between 30 and 300 Mbps showed a more stable association, which is consistent with the everyday digital needs of many older adults, including health information access, video calls, essential online services, and social communication. The marginal relevance of ultra-high-speed connectivity therefore appears more group-specific than universal.

To quantify uncertainty and support interpretation of high-impact variables, bootstrap confidence intervals and SHAP summaries were added. In the low-social-status group, gigabit availability had a coefficient of -0.24 with a 95% bootstrap CI of -0.33 to -0.15 and a mean absolute SHAP value of 0.082. Average download speed had a coefficient of -0.21, 95% CI -0.30 to -0.12, and mean absolute SHAP value 0.076. In the high-deprivation group, 30-300 Mbps coverage had a coefficient of -0.19, 95% CI -0.28 to -0.09, and mean absolute SHAP value 0.071. Across-group comparisons of bootstrap coefficient distributions showed significant differences for gigabit availability (adjusted *p* = 0.018), average download speed (adjusted *p* = 0.031), 30-300 Mbps coverage (adjusted *p* = 0.044), and low-speed connections below 30 Mbps (adjusted *p* = 0.027).

### 3.4. Spatial contributions and key areas of identification

**Figure 5.**
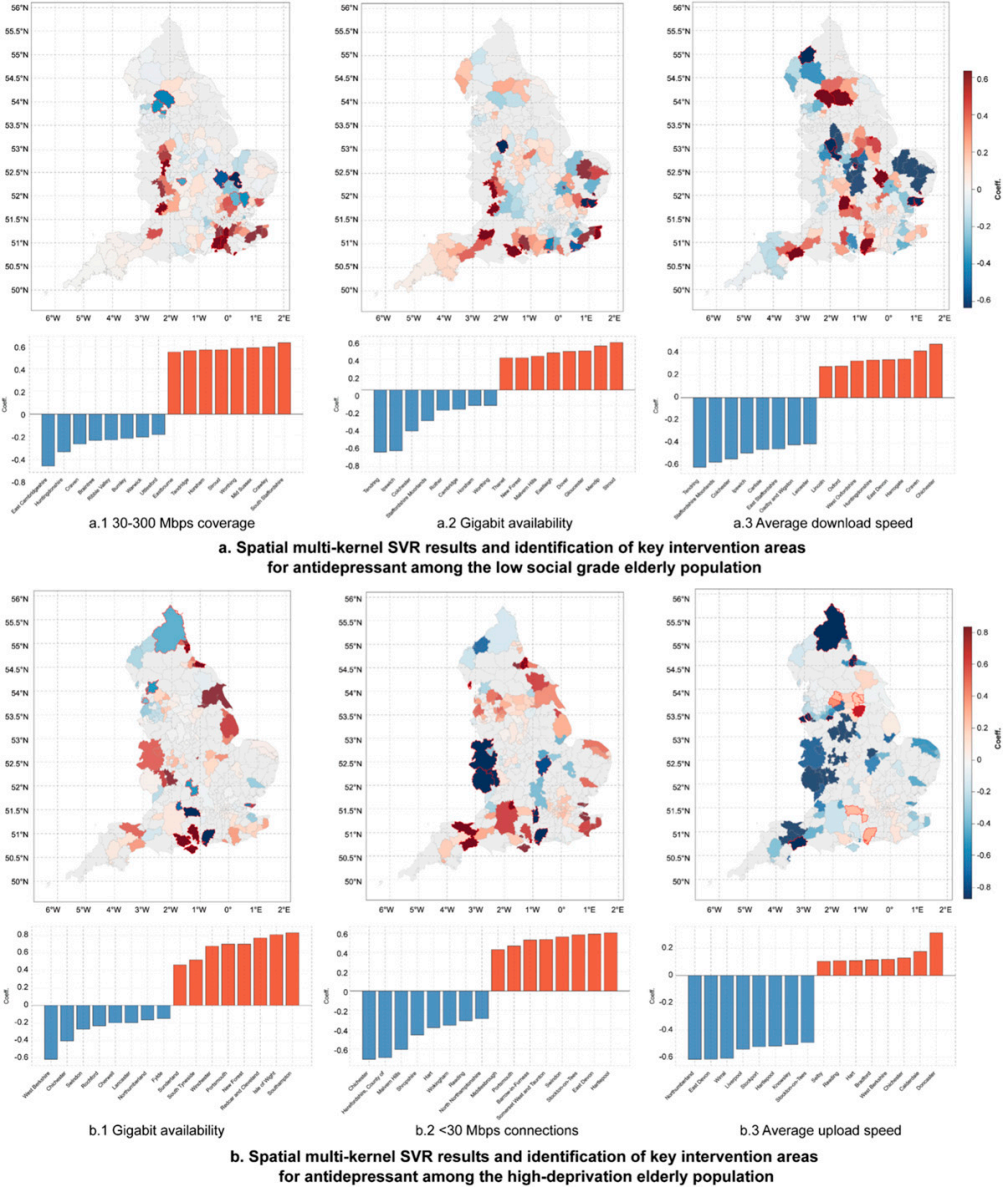
Spatial multi-kernel SVR maps pinpointing digital-infrastructure priority areas for elderly mental health in low social grade group (a) and high-deprivation group (b).

The spatially weighted multi-kernel SVR analysis identifies geographically varying associations between digital infrastructure and depression-related burden. Figure 5 presents the spatial distributions of the regression coefficients for six digital-infrastructure variables in the two target groups. Rather than treating these maps as causal intervention effects, the study interprets them as policy-relevant priority patterns that identify where broadband quality is most strongly associated with depression-related burden after built-environment covariates are considered.

For the low-social-status group (Figure 5(a)), the spatial coefficients of 30-300 Mbps medium-speed broadband coverage show a north-south contrast. The Northwest, southern parts of the East Midlands, and northern parts of the Southeast display stronger negative coefficients, indicating areas where improved medium-speed broadband coverage is associated with lower depression-related burden. These areas include post-industrial or economically transitioning communities where DE social grade populations are more concentrated. In contrast, parts of the London commuter belt and the West Midlands show weaker or positive coefficients, suggesting that broadband coverage alone may have lower marginal relevance in places where other service and infrastructure conditions differ.

For the high-deprivation older population (Figure 5(b)), the spatial patterns point to a different set of priority geographies. Gigabit availability shows stronger negative coefficients in remote and ageing communities in the North East, Northumberland and Durham, parts of Lancashire, and areas of the Southwest. Low-speed connections below 30 Mbps show positive coefficients in parts of the West Midlands, Yorkshire, and Somerset, indicating that reliance on lower-quality connectivity is associated with higher depression-related burden in some ageing and service-constrained areas. Upload speed also appears relevant in places where older residents may rely on video communication, telehealth, and digital social interaction. These findings support differentiated policy interpretation: post-industrial communities may require high-quality broadband investment, whereas remote older communities may benefit from stable medium-speed coverage, upload capacity, and service-oriented digital support.

## 4. Discussion

### 4.1. Conceptual contributions and methodological innovation

The core conceptual contribution of this study is the digital quality gradient framework. This framework moves beyond the binary distinction between being connected and unconnected by considering broadband quality as an ordered spectrum from low-speed (*<*30 Mbps) to medium-speed (30-300 Mbps), high-speed (≥300 Mbps), gigabit availability, and observed download and upload performance. The results suggest that broadband quality is associated with depression-related burden in different ways across vulnerable groups. The framework therefore provides a way to interpret digital infrastructure as part of the contemporary built environment, while avoiding the assumption that all types of Internet access are equivalent for mental-health-related digital use.

Methodologically, the study contributes an area-level modelling framework that integrates stepwise regression, multi-kernel SVR, and spatial weighting. The stepwise models make the relationship between built-environment and digital-infrastructure covariates transparent. The multi-kernel SVR extends this analysis by allowing linear, local nonlinear, and threshold-like patterns to be captured within a single predictive model. The spatial weighting layer then adds a local interpretation of where digital-infrastructure variables show stronger or weaker associations with depression-related burden. This combination is particularly suited to tabular, spatially aggregated public health data, where predictive performance, nonlinear flexibility, and policy interpretability need to be balanced.

The analysis also clarifies the role of the built environment. Building counts, POI categories, and POI diversity were retained as contextual covariates because they represent conventional urban structure, service access, and opportunities for social participation. These variables showed more stable and less spatially heterogeneous coefficient patterns than the digital-infrastructure variables. Therefore, the built environment should not be interpreted as irrelevant or dissolved in the model; instead, it provides the physical and service context within which the added predictive contribution of broadband quality can be interpreted. This interpretation strengthens the integrated infrastructural framing of the study.

The policy contribution is the identification of priority geographies from spatial coefficient patterns. These maps should be interpreted as evidence of spatially varying associations rather than direct causal intervention effects. They show where digital infrastructure is most strongly associated with depression-related burden after built-environment covariates are considered. In practical terms, this supports a differentiated approach to digital-health equity: post-industrial communities with low social grade concentrations may require high-quality broadband investment, while remote and ageing communities may require stable medium-speed coverage, upload capacity, and service-oriented digital support.

### 4.2. Key findings and policy implications for digital mental health equity

This study used a multi-stage modelling approach to examine how digital infrastructure is associated with depression-related burden among older adults in socio-economically vulnerable areas. The results indicate that digital infrastructure is a relevant area-level predictor, but its relevance differs by vulnerable group. In high-deprivation older areas, the addition of digital variables moderately improved model fit after built-environment covariates were included. In low-social-status areas, digital infrastructure variables showed stronger predictive contribution, particularly for broadband quality indicators. These findings support the argument that broadband quality is not only a technical infrastructure issue, but also a component of spatial inequality in digital health access.

The coefficient distributions and validation results support a cautious nonlinear interpretation. The multi-kernel SVR performed better than linear and regularized linear baselines in cross-validation, and the bootstrap and SHAP summaries identify several high-impact digital-infrastructure variables. For low-social-status areas, gigabit availability, high-speed connections, and average download speed showed stronger predictive contributions. For high-deprivation older areas, medium-speed coverage and upload-related capacity appeared more consistently relevant. This distinction helps explain why a single broadband policy measure may not suit all vulnerable groups.

The spatially weighted model also shows that regional interpretation matters. Post-industrial districts in Northern England and the Midlands, including areas around Manchester, Liverpool, Birmingham, and Yorkshire, appear as important policy-relevant geographies for low-social-status populations. Remote, coastal, and ageing communities in the North East, Northumber-land, Durham, Lancashire, Somerset, and parts of the Southwest show different digital-infrastructure needs for high-deprivation older populations. These regional characterizations are now included to help non-local readers interpret the maps.

Policy implications are therefore presented in calibrated terms. First, digital-health equity should consider broadband quality, not only basic access. Second, digital infrastructure should be planned together with conventional built-environment and service-access conditions, because broadband can support telehealth, online social support, and health-information access only when residents can use those services effectively. Third, spatial coefficient maps can help identify where further local assessment and targeted investment may be most useful. The results do not by themselves establish causal intervention effects, but they provide an evidence base for prioritizing places where broadband quality and depression-related burden are most strongly associated.

### 4.3. Strengths and limitations

This study has several strengths. It combines built-environment and digital-infrastructure variables within the same modelling framework, defines vulnerable groups using both older-age income deprivation and DE social grade, and evaluates nonlinear and spatially heterogeneous associations using multi-kernel SVR and spatial weighting. The study demonstrates high reproducibility through robust model validation, hyperparameter configurations and uncertainty estimates.

Several limitations should also be acknowledged. First, the cross-sectional ecological design prevents causal inference between digital infrastructure and mental health. These findings should therefore be interpreted as area-level associations and predictive spatial patterns. Second, the use of LSOA-level aggregated data may lead to ecological fallacy, because within-area variation in digital literacy, usage habits, health needs, and individual social networks is not observed. Third, the study measures objective infrastructure availability and performance rather than individual-level digital capability or actual use of online health services. Fourth, although multi-kernel SVR and SHAP summaries improve nonlinear interpretation, they do not replace longitudinal, quasi-experimental, or individual-level evidence. Future research should combine infrastructure data with individual survey data, digital-skills measures, and longitudinal broadband rollout designs to test the mechanisms suggested by the present spatial patterns.

## 5. Conclusions and future work

This study examined area-level associations between digital infrastructure and depression-related burden among socio-economically vulnerable older populations in England. Using LSOA-level spatial data, the study combined stepwise regression, multi-kernel SVR, and spatially weighted modelling to evaluate whether broadband quality adds predictive information after conventional built-environment covariates are considered. The analysis focused on two vulnerable groups: areas with high proportions of DE social grade residents and areas with high income deprivation among older adults. By defining a digital quality gradient across broadband speed tiers, gigabit availability, connection counts, and observed download/upload performance, the study provides a spatial framework for examining how digital infrastructure is associated with later-life mental-health inequality.

The findings show that digital-infrastructure variables have different predictive patterns across the two vulnerable groups. In low-social-status areas, broadband quality indicators such as gigabit availability, high-speed connections, and average download speed made stronger contributions to model performance than conventional built-environment variables alone. In high-deprivation older areas, medium-speed coverage and upload-related capacity showed more consistent relevance, suggesting that the digital needs of older residents may differ from those of broader socio-economically disadvantaged populations. The multi-kernel SVR model achieved stronger cross-validated performance than linear baselines, supporting the use of nonlinear modelling for this type of spatial health analysis.

The study makes three main contributions. Conceptually, it introduces the digital quality gradient as a way to move beyond binary measures of Internet access. Methodologically, it shows how multi-kernel SVR and spatial weighting can be combined to examine nonlinear and geographically heterogeneous area-level associations. For policy interpretation, it identifies priority geographies where broadband quality and depression-related burden are most strongly associated, including post-industrial communities in the North and Midlands and remote or ageing communities in northern and southwestern England.

These conclusions should be interpreted within the limits of a cross-sectional ecological study. The results do not establish causal mechanisms or individual-level health effects. Future research should combine area-level infrastructure data with individual-level digital-skills and health-service-use data, and should use longitudinal or quasi-experimental designs based on broadband rollout to test the mechanisms suggested by the present spatial patterns.

## Supplemental material

Supplemental material - Digital quality gradient and the burden of elderly depression: A spatial multi kernel SVR analysis of socio economically vulnerable groups in England Supplemental material for Digital quality gradient and the burden of elderly depression: A spatial multi kernel SVR analysis of socio economically vulnerable groups in England by in Rui, Yijing Li, Hector D. Menendez, Mariana Pinto da Costa in Digital Health.

## Data Availability

The study used area-level data from publicly available and licensed na-tional datasets, including the Ofcom Connected Nations Spring 2025 fixed broadband data, the Small Area Mental Health Indices (SAMHI), Office for National Statistics population data, Ordnance Survey Open Data, and Ordnance Survey Points of Interest data accessed through Digimap. Derived variables and intermediate processing outputs can be made available by the corresponding author upon reasonable request, subject to the licensing conditions of the original data providers.*
