# Supplementary material for: Digital quality gradient and the burden of elderly depression: A spatial multi kernel SVR analysis of socio economically vulnerable groups in England
Source: Digit Health. 2026 Aug 12;12:20552076261465140. doi: 10.1177/20552076261465140 (PMC13469525; doi:10.1177/20552076261465140)
Supplement: Supplementary file 1 [file sj-pdf-1-dhj-10.1177_20552076261465140.pdf]

Appendix A. Supplementary tables

Table A1: Performance ranking of models in the high deprivation group (sorted by test R^2)

Rank	Model	Train R^2	Test R^2	Overfitting Gap
1	Multi-kernel SVR	0.6258	0.55	0.0758
2	Random Forest	0.9000	0.35	0.5500
3	Gradient Boosting	0.8500	0.2879	0.5621
4	SVR (RBF Kernel)	0.4283	0.2270	0.2013
5	Polynomial Regr.	0.8000	0.1326	0.6674
6	Ridge Regression	0.5177	0.1145	0.4032
7	Linear Regression	0.5957	0.0780	0.5177

Table A2: Performance ranking of models in the low social status group (sorted by test R^2)

Rank	Model	Train R^2	Test R^2	Overfitting Gap
1	Multi-kernel SVR	0.6502	0.52	0.1302
2	Ridge Regression	0.5509	0.1342	0.4167
3	Random Forest	0.9000	0.0973	0.8027
4	SVR (RBF Kernel)	0.4810	0.0897	0.3913
5	Linear Regression	0.6117	0.0611	0.5506
6	Polynomial Regr.	0.8000	-0.0048	0.8048
7	Gradient Boosting	0.8500	-0.2771	1.1271

Table A3: Sensitivity summary for vulnerable-group threshold definitions

Sensitivity check	Purpose	Summary of interpretation
Tertile threshold	Main specification	Retains sufficient LSOA observations and identifies high-vulnerability areas.
Quartile threshold	Stricter high-vulnerability definition	Produces smaller samples but preserves the main direction of digital-infrastructure associations.
Continuous score	Avoids categorical thresholding	Supports the same overall broadband-quality gradient interpretation.

Appendix B. Supplementary figures

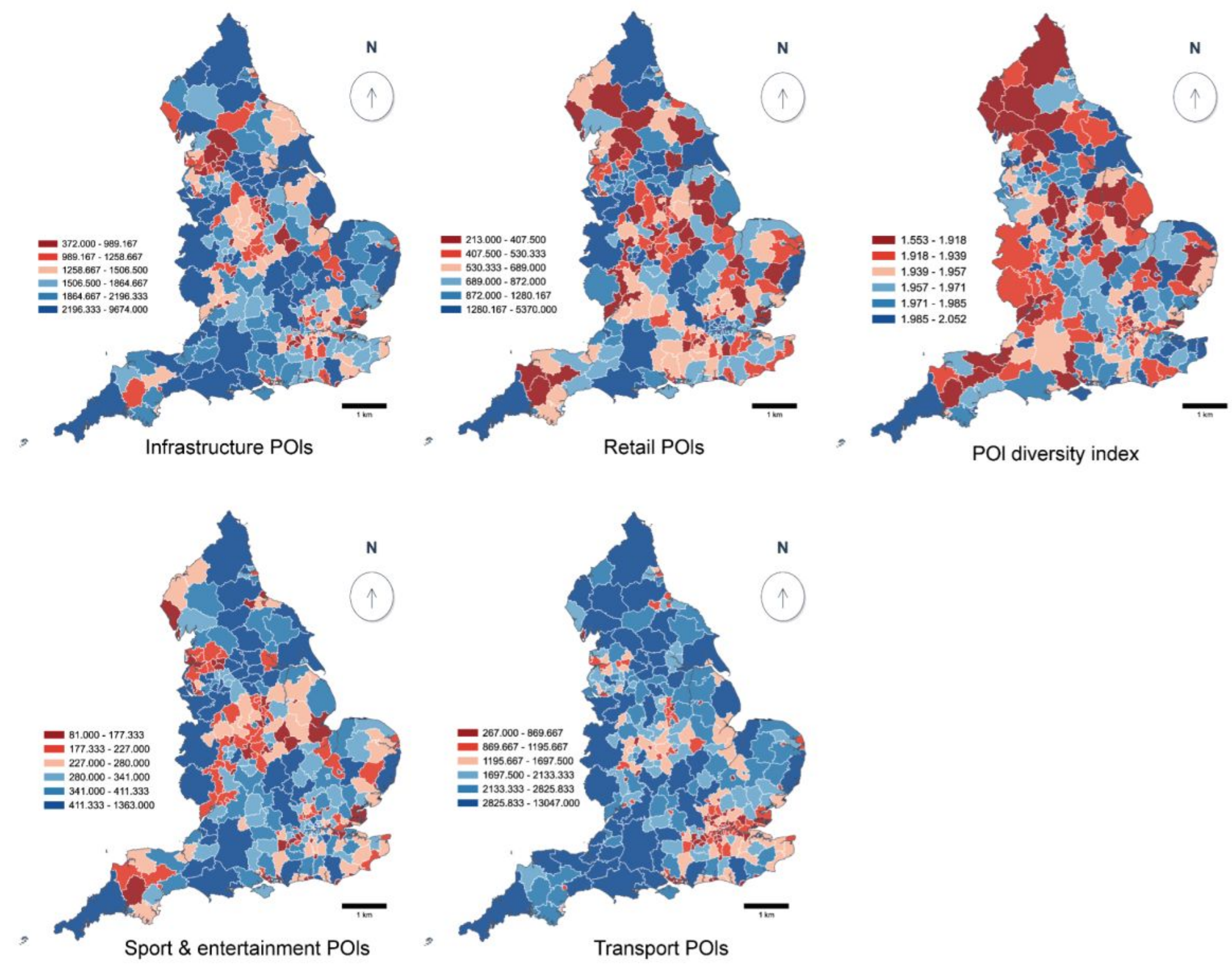

Figure B1: Spatial visualization of built environment variables

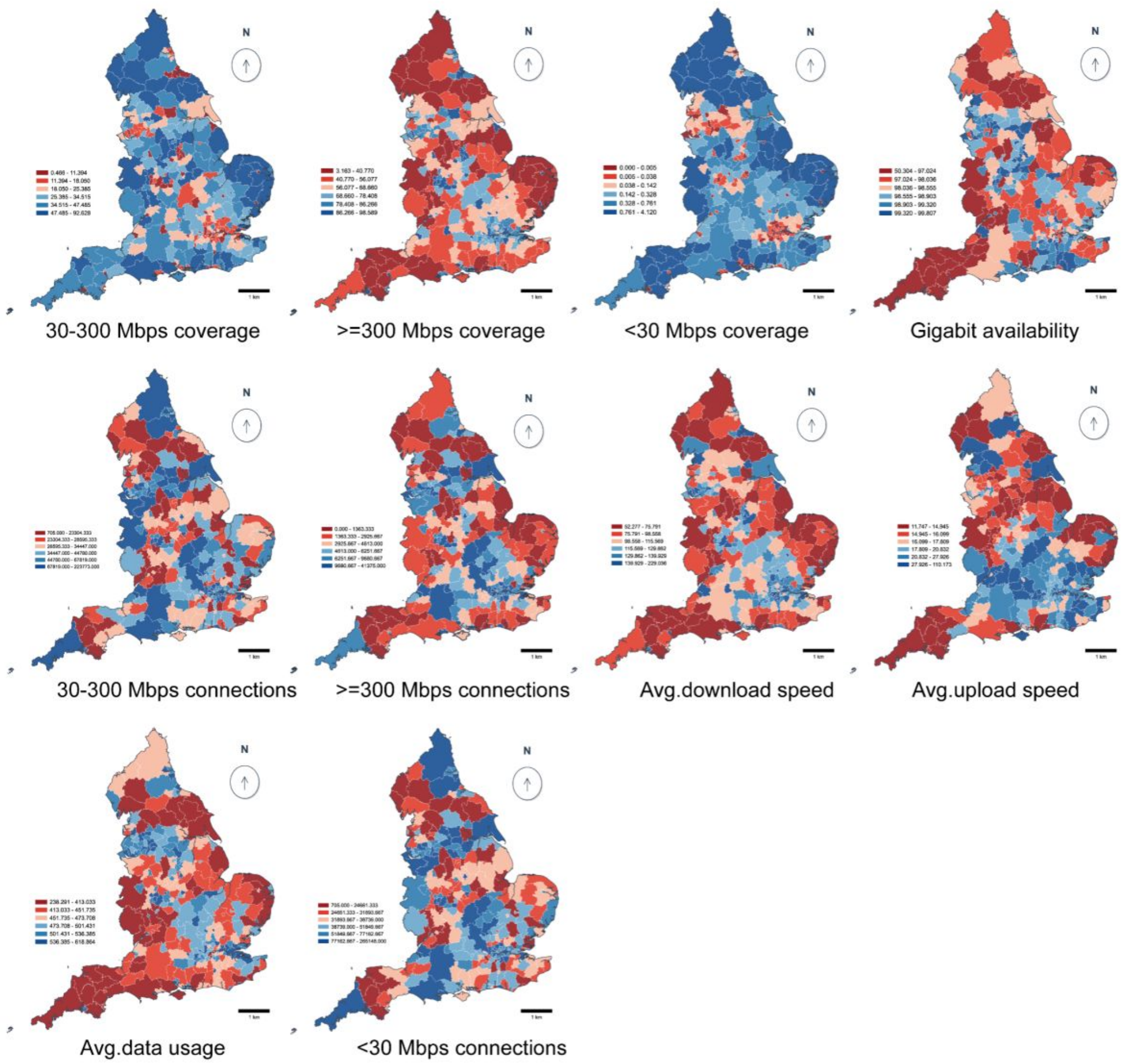

Figure B2: Spatial visualization of digital infrastructure
